# Solitary Pulmonary Mass due to *Mycobacterium avium* Infection in a Post‐Traumatic Lung: A Case Report

**DOI:** 10.1002/rcr2.70543

**Published:** 2026-03-10

**Authors:** Takayuki Nakano, Shunsuke Yamada, Kozo Morimoto, Saya Hattori, Rino Arai, Mayumi Aoyama, Hideki Hashimoto, Tomoki Nakagawa, Hidenobu Shigemitsu, Ryota Masuda, Ichiro Kuwahira

**Affiliations:** ^1^ Respiratory Disease Center Tokyo General Hospital Tokyo Japan; ^2^ Department of General Thoracic Surgery Tokai University Hachioji Hospital Tokyo Japan; ^3^ Division of General Thoracic Surgery, Department of Surgery Tokai University School of Medicine Kanagawa Japan; ^4^ Respiratory Disease Center, Fukujuji Hospital, Japan Anti‐Tuberculosis Association Tokyo Japan; ^5^ Roseman University School of Medicine Las Vegas, Nevada USA

**Keywords:** *Mycobacterium avium*, post‐traumatic lung, pulmonary NTM disease, solitary pulmonary mass, surgical resection

## Abstract

Solitary pulmonary nodule or mass like presentation of pulmonary *Mycobacterium avium* complex (MAC) disease is uncommon. We report a case of solitary pulmonary mass caused by MAC infection arising in a post‐traumatic lung. A 56‐year‐old man with a history of left chest trauma, including pulmonary contusion and traumatic pneumatocele in the left lower lobe (LLL), was found to have a solitary mass in the same lobe. Chest computed tomography revealed a 33‐mm subpleural mass in the LLL. Bronchoscopy was non‐diagnostic. Serum anti‐glycopeptidolipid‐core IgA antibody was positive, but bronchoalveolar lavage culture and polymerase chain reaction (PCR) were negative. Thoracoscopic partial resection of the LLL was performed for diagnosis and treatment. Histopathology revealed epithelioid granulomatous inflammation with necrosis, and culture and PCR confirmed *Mycobacterium avium* infection. The postoperative course was uneventful, and the patient has been managed conservatively without additional antimicrobial therapy. To our knowledge, this is the first case of MAC infection arising in a post‐traumatic lung as a solitary pulmonary mass.

## Graphical abstract

We report a case of solitary nodular *Mycobacterium avium* infection arising in a post‐traumatic lung. Chest CT showed a solid mass in the left lower lobe, and the diagnosis was established through thoracoscopic surgical resection. Graphical abstract image Figure 2C,D.

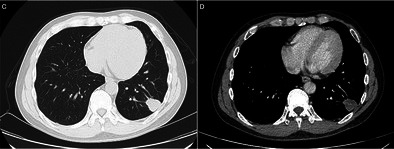

## 1 Introduction

The incidence of pulmonary nontuberculous mycobacterial (NTM) disease has been increasing, with *Mycobacterium avium* complex (MAC) being the most common causative organism [1, 2, 3]. Pulmonary NTM disease rarely presents as a solitary pulmonary nodule or mass and may be difficult to distinguish from lung cancer [3, 4, 5].

## 2 Case Report

**FIGURE 1:**
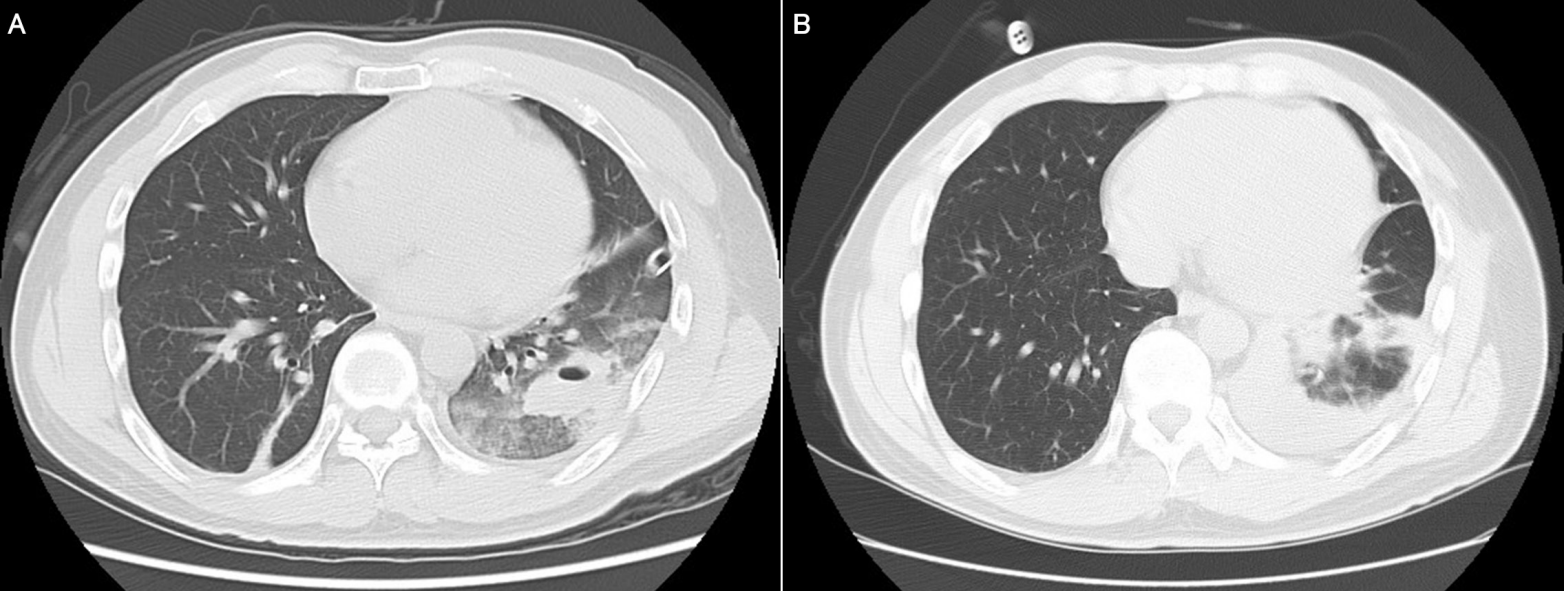
(A) Chest CT at the time of the first accident (10 years before presentation) showing pulmonary contusion and traumatic pneumatocele in the left lower lobe. No significant traumatic changes were observed in the other lobes. (B) Chest CT at the time of the second accident (6 months after the first accident) showing left hemothorax and subpleural infiltrates in the left lower lobe, while the previous pulmonary contusion and traumatic pneumatocele had resolved, with no significant abnormalities in the other lobes.

**FIGURE 2:**
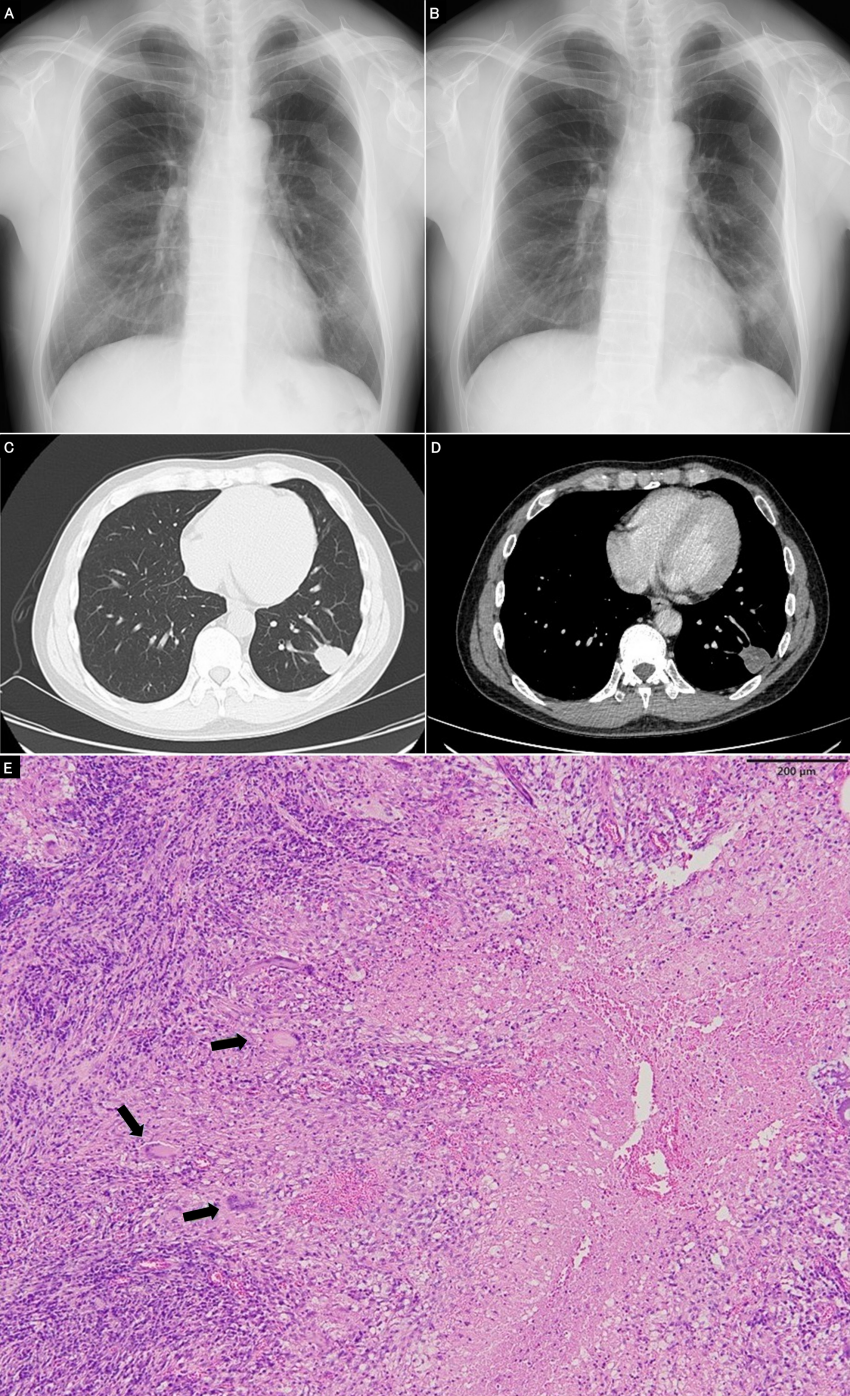
(A) Chest radiograph obtained 4 years before presentation (6 years after chest trauma). (B) Chest radiograph at presentation (10 years after chest trauma) showing enlargement of the abnormal shadow in the left lower lung field. (C) Current chest CT showing a solitary mass in the subpleural region of the left lower lobe. (D) Contrast‐enhanced CT showing a mixture of enhancing and low‐attenuation areas. (E) Histopathological examination revealed epithelioid granulomas with necrosis and scattered Langhans‐type giant cells (arrows). No trauma‐related changes were identified. Haematoxylin and eosin stain, ×100. Scale bar = 200 μm.

A 56 year‐old man was found to have an enlarging opacity in the left lower lung field (LLLF) on chest radiograph during a routine health checkup. His only comorbidity was dyslipidemia and a history of smoking 5 cigarettes daily between age 15 and 40. The patient was an avid swimmer, triathlete, and motorcycle enthusiast. He had sustained two left chest traumas from prior motorcycle accidents. The first accident occurred 10 years ago, resulting in multiple left rib fractures, pulmonary contusion, traumatic pneumatocele in the left lower lobe (LLL), and traumatic hemopneumothorax on chest computed tomography (CT) (Figure 1A). He was treated conservatively with chest tube drainage. Six months later, the patient suffered a second accident which resulted in a left traumatic hemothorax that was managed conservatively. Chest CT demonstrated subpleural infiltrates in the LLL; however, the previously noted pulmonary contusion and pneumatocele had improved, and no significant traumatic changes were observed in the other lobes (Figure 1B). Six years after this trauma, a new abnormal shadow was detected in the LLLF on a chest radiograph, which was found to have enlarged at the time of the current presentation (Figure 2A,B).

At the time of evaluation, the patient was asymptomatic. Physical examination revealed a middle‐aged man that was 173 cm tall, weighed 67 kg (body mass index [BMI] 22.4 kg/m^2^), and had an unremarkable examination. Laboratory studies including tumour markers were normal. Serum anti‐glycopeptidolipid (GPL)‐core IgA antibody was positive at 1.36 U/mL (cut‐off value: 0.7 U/mL), and interferon‐gamma release assay (IGRA) was negative. Chest CT showed a 33‐mm solid mass in the subpleural LLL without internal calcification, containing both enhancing and low‐attenuation areas (Figure 2C,D). Bronchoscopy revealed no malignant or granulomatous findings. Bronchoalveolar lavage culture and mycobacterial polymerase chain reaction (PCR) testing were negative. Although the positive anti‐GPL‐core IgA antibody raised suspicion for pulmonary MAC disease, the atypical presentation as a solitary mass and negative bronchoscopic findings prevented definitive preoperative diagnosis.

Image‐guided biopsy was not performed due to concerns about sampling only necrotic material given the mixed enhancing and low‐attenuation areas on CT, and the patient's strong preference for surgical resection. Fluorodeoxyglucose positron emission tomography (FDG‐PET) was not performed because FDG‐PET is not covered by health insurance in Japan for benign‐malignant differentiation, and FDG‐PET has limited specificity as it shows uptake in both malignant and inflammatory lesions.

Since malignancy could not be excluded, thoracoscopic partial resection of the LLL was performed. Histopathological examination revealed epithelioid granulomatous inflammation with necrosis, without evidence of malignancy (Figure 2E). Culture and PCR were positive for *Mycobacterium avium* ( *M. avium* ), confirming pulmonary *M. avium* infection. The postoperative course was uneventful, and no additional antimicrobial therapy was given. At 6 months postoperatively, the patient remained asymptomatic, with no new lesions identified on CT.

## 3 Discussion

We report a case of MAC disease presenting as a solitary pulmonary mass in a post‐traumatic lung in a middle‐aged man with an average BMI who was an avid swimmer with no underlying diseases other than chest trauma.

The incidence of pulmonary NTM disease has increased markedly worldwide. In Japan, MAC accounts for approximately 90% of cases [2]. NTM organisms are ubiquitous in water and soil, and environmental exposures such as aerosols from showerheads and indoor swimming pools are considered potential sources of infection [2]. However, not all individuals exposed to NTM develop pulmonary disease, suggesting that interplay between the organism and host factors are essential in disease susceptibility [2]. Pulmonary NTM disease is more common in people with pre‐existing structural lung abnormalities, such as chronic obstructive pulmonary disease (COPD) and bronchiectasis [2]. Structural damage to the airways can reduce mucociliary clearance, increasing susceptibility to NTM infection [2].

Pulmonary NTM disease is typically classified into fibrocavitary and nodular bronchiectatic types based on radiologic patterns, whereas presentation as a solitary pulmonary nodule or mass is uncommon and often mimics lung cancer [3, 4, 5]. A previous report indicated that solitary pulmonary nodule or mass presentation accounted for 3.6% of pulmonary NTM cases [4].

According to the 2020 ATS/ERS/ESCMID/IDSA guideline, diagnosis of pulmonary NTM disease requires assessment of clinical, radiographic, and microbiological criteria [1]. However, in asymptomatic patients with a solitary nodule or mass suspected to be caused by NTM infection, these criteria are often not fully satisfied, and surgical resection is frequently required to establish a definitive diagnosis [3].

Structural lung abnormalities are well recognised as a risk factor for NTM disease [1, 2]. Previous reports have described NTM lesions not only in patients with COPD and bronchiectasis, but also in structural changes secondary to hypoperfusion in chronic thromboembolic pulmonary hypertension (CTEPH) [6] and at surgical resection margins. However, to our knowledge, no cases of solitary nodule or mass from NTM infection arising in a post‐traumatic lung have been reported.

The lesion in our case appeared exclusively in the subpleural area of the LLL, where previous trauma had been confirmed. This anatomical concordance suggests that the association is unlikely to be explained by coincidence alone. We propose the following pathophysiological mechanisms by which post‐traumatic lung changes may have predisposed to NTM infection. First, structural changes in the lung parenchyma caused by trauma may lead to impaired mucociliary clearance. Blunt chest trauma can cause microscopic bronchial epithelial damage, localised fibrosis, and microatelectasis. Even when these changes are not visible on histopathology years after injury, they may persist at a microscopic level and result in impaired bacterial clearance mechanisms, creating a local environment susceptible to NTM colonisation. Second, it is well established that structural lung disease increases susceptibility to NTM infection [2], and there are reports of NTM disease occurring in lungs with ventilation‐perfusion abnormalities such as in CTEPH [6]. Third, NTM bacteria have frequently been detected in indoor swimming pools [7, 8, 9], which are recognised as an important source of NTM exposure [2, 7, 8, 9]. Our patient's regular swimming activity may have led to repeated NTM exposure through inhalation of contaminated water aerosols. Although we cannot entirely exclude the possibility of direct bacterial inoculation during the traumatic event or subsequent chest tube placement, epidemiological studies support that the primary route of pulmonary NTM infection is via inhalation of aerosolized organisms rather than direct inoculation. The time interval between trauma and lesion detection (approximately 6 years) also suggests chronic infection following repeated inhalational exposure rather than acute inoculation at the time of injury.

Although histopathological examination did not demonstrate trauma‐related changes in the resected lesion, the solitary MAC lesion developed in the LLL, corresponding to the site of prior injury. We propose that the combination of localised post‐traumatic structural vulnerability (impaired mucociliary clearance and altered local immunity) and repeated environmental NTM exposure from indoor swimming pools created conditions favourable for NTM colonisation, infection, and proliferation through the interaction between host and environmental factors described in the literature [2].

Although MAC disease was not diagnosed preoperatively, surgical resection was nonetheless appropriate given the limited efficacy of antimicrobial therapy for NTM disease, the solitary lesion, and the feasibility of complete resection. The optimal approach to NTM disease presenting as a solitary pulmonary nodule or mass is uncertain [3]. The 2020 ATS/ERS/ESCMID/IDSA guideline advises surgery only as an adjunctive therapy in selected patients [1]. However, several case series have reported that patients with NTM presenting as a solitary pulmonary nodule or mass were successfully treated with resection alone [5]. This case highlights the importance of considering NTM disease in the differential diagnosis of solitary pulmonary nodule or mass and illustrates that surgical resection can provide both definitive diagnostic and therapeutic benefit.

## Author Contributions

T.N. and S.Y. performed the surgical procedure. S.H., R.A., M.A., H.H. and I.K. performed the bronchoscopy. T.N. drafted the manuscript. S.Y., K.M., T.N., H.S., R.M. and I.K. critically revised the manuscript for important intellectual content. All authors read and approved the final version of the manuscript.

## Consent

The authors declare that written informed consent was obtained for the publication of this manuscript and accompanying images using the consent form provided by the Journal.

## Conflicts of Interest

Kozo Morimoto is an Editorial Board member of Respirology Case Reports and a co‐author of this article. He was excluded from all editorial decision‐making related to the acceptance of this article for publication. The other authors have no conflicts of interest to declare.

## Data Availability

Research data are not shared.
